# Epidemiological and clinical characteristics of imported cases of COVID-19: a multicenter study

**DOI:** 10.1186/s12879-021-06096-6

**Published:** 2021-05-03

**Authors:** Jinjun Zhang, Shixiong Yang, Yi Xu, Xinyan Qin, Jialiang Liu, Jinjun Guo, Sijia Tian, Shaoping Wang, Kai Liao, Ying Zhang, Yu Ma, Yuguo Chen

**Affiliations:** 1 School of Medicine, Shandong University, Jinan, Shandong 250012 China; 2 Beijing Emergency Medical Center, Beijing, 100031 China; 3 Nanning Emergency Medical Center, Nanning, Guangxi 530022 China; 4 Chongqing University Central Hospital, Chongqing Emergency Medical Center, Chongqing, 630014 China; 5 Uninted Family Healthcare, Beijing, 100015 China; 6 Jinan Emergency Medical Center, Jinan, Shandong 250021 China; 7 Emergency Department, Qilu Hospital, Shandong University, Shandong Provincial Clinical Research Center for Emergency and Critical Care Medicine, Jinan, Shandong 250012 China

**Keywords:** COVID-19, Epidemiological characteristics, Clinical feature; imported case

## Abstract

**Background:**

The coronavirus disease 2019 (COVID-19) pandemic continues to expand. Herein, we report the epidemiological and clinical features of 478 patients with confirmed COVID-19 from a multicenter study conducted in four cities in China excluding Wuhan.

**Methods:**

A total of 478 patients transferred by emergency medical services to designated hospitals in four major cities in China (Beijing, Chongqing, Jinan, and Nanning) were enrolled. We compared the characteristics of imported and indigenous cases and calculated the frequencies of fatal, severe, mild, and asymptomatic disease. The results were used to generate a pyramid of COVID-19 severity.

**Results:**

The mean age of patients with COVID-19 was 46.9 years and 49.8% were male. The most common symptoms at onset were fever (69.7%), cough (47.5%), fatigue (24.5%), dyspnea (8.4%), and headache (7.9%). Most cases (313, 65.5%) were indigenous, while 165 (34.5%) were imported. Imported cases dominated during the early stages of the pandemic, but decreased from 1 February 2020 as indigenous cases rose sharply. Compared with indigenous cases, imported cases differed significantly in terms of sex (*P* = 0.002), severity of disease (*P* = 0.006), occurrence of fever (*P* < 0.001), family clustering (*P* < 0.001), history of contact (*P* < 0.001), and primary outcome (*P* < 0.001).

**Conclusions:**

Within the population studied, imported cases had distinct characteristics from those of indigenous cases, with lower fatality rates and higher discharge rates. New infections shifted from imported cases to local infection gradually, and overall infections have declined to a low level. We suggest that preventing import of cases and controlling spread within local areas can help prevent SARS-CoV-2 infection spread.

## Background

According to a World Health Organization (WHO) report, three million cases of laboratory-confirmed severe acute respiratory syndrome coronavirus-2 (SARS-CoV-2) infection and the resulting syndrome, termed coronavirus disease 2019 (COVID-19), had been detected globally as of 30 April 2020 [1, 2]. In mainland China, 81,518 patients were confirmed to have COVID-19, among whom 3305 have died, 76,052 have been discharged, and 2161 remain hospitalized [3]. Confirmed cases of COVID-19 have also been reported in Italy, Thailand, Singapore, Vietnam, the United States, and more than 200 other countries and regions globally [4–6]. Studies of the clinical features of COVID-19 published early in the pandemic mainly focused on Wuhan, China [7–10]. The data were obtained from the early stages of the outbreak in Wuhan, when reported cases might have been incompletely observed and patients may have been admitted to intensive care units following publication. Because COVID-19 now represents a global public health issue, it is understandable that its spread is causing confusion, anxiety, and fear among members of the public.

Several single-center studies have described the clinical characteristics and epidemiology of imported cases of COVID-19 in China [11, 12]. Because local SARS-CoV-2 infection often results from unobserved imported cases with high infectivity [13], we hypothesized that imported cases of COVID-19 would have distinct epidemiological and clinical features compared with indigenous cases. Emergency medical service (EMS) crews typically include a physician, a nurse, and a driver; these crews are responsible for transporting confirmed cases of COVID-19 from the community, hospitals, or quarantine locations to designated hospitals during the epidemic in China. The purpose of this multicenter study was to analyze the epidemiological and clinical characteristics of imported cases with confirmed COVID-19 transferred by EMS in four cities outside of Wuhan, China. We hoped to unravel the relationships between imported and indigenous cases of COVID-19.

## Methods

### Study design

We retrospectively analyzed patients diagnosed with COVID-19 who were transferred by EMS from 20 January 2020 to 20 February 2020 in four cities in China (Beijing, Chongqing, Jinan, and Nanning). None of these cities are near Wuhan. Only patients with laboratory-confirmed SARS-CoV-2 infection were included in this study. Patients with confirmed infections who were not transported by EMS were not included. The study was approved by the Ethics Committee of the Beijing Emergency Medical Center (No. 2020–01) and the requirement for written informed consent was waived.

### Study definitions

The definition of a confirmed case of COVID-19 was based on positive viral nucleic acid tests from throat swabs according to the new coronavirus pneumonia diagnosis and treatment program. Patients with asymptomatic infection were defined as confirmed cases who were afebrile and well. Patients with mild infection were defined as confirmed cases with fever, respiratory symptoms, and radiographic evidence of pneumonia. Patients with severe infection were defined as confirmed cases with dyspnea (respiratory frequency ≥ 30/min, blood oxygen saturation ≤ 93%) or respiratory failure [14]. Imported cases were defined as patients with confirmed COVID-19 who acquired SARS-CoV-2 infection outside the area where it was diagnosed, as per the WHO definition of imported cases [15]. This definition was made possible by epidemiologic investigation of patients’ travel histories to Hubei province, especially to the Wuhan endemic area. Indigenous cases were defined as patients with confirmed COVID-19 who were infected within local areas.

### Data sources

Data included demographic information, epidemiological characteristics, clinical data, laboratory test results for SARS-CoV-2, diagnostic test type, case clustering, and outcome. Data were collected by EMS providers in four major cities in China. Clinical outcomes were followed up until 20 February 2020. If data were missing from medical records, they were obtained by direct communication with EMS providers.

### Statistical analysis

Continuous variables were expressed as means and standard deviations. Mann–Whitney U test was used to compare respiratory rate with the assumption of unequal variance (D = 0.429, *P* < 0.001). Categorical variables were presented as counts and percentages. Differences between categorical variables between imported and indigenous cases were assessed using the chi-square test. All statistical analyses were performed using SPSS software version 22.0 (IBM, Armonk, NY, USA). Values of *P* < 0.05 were considered statistically significant.

## Results

### Demographic and clinical features of COVID-19 patients

**Fig. 1:**
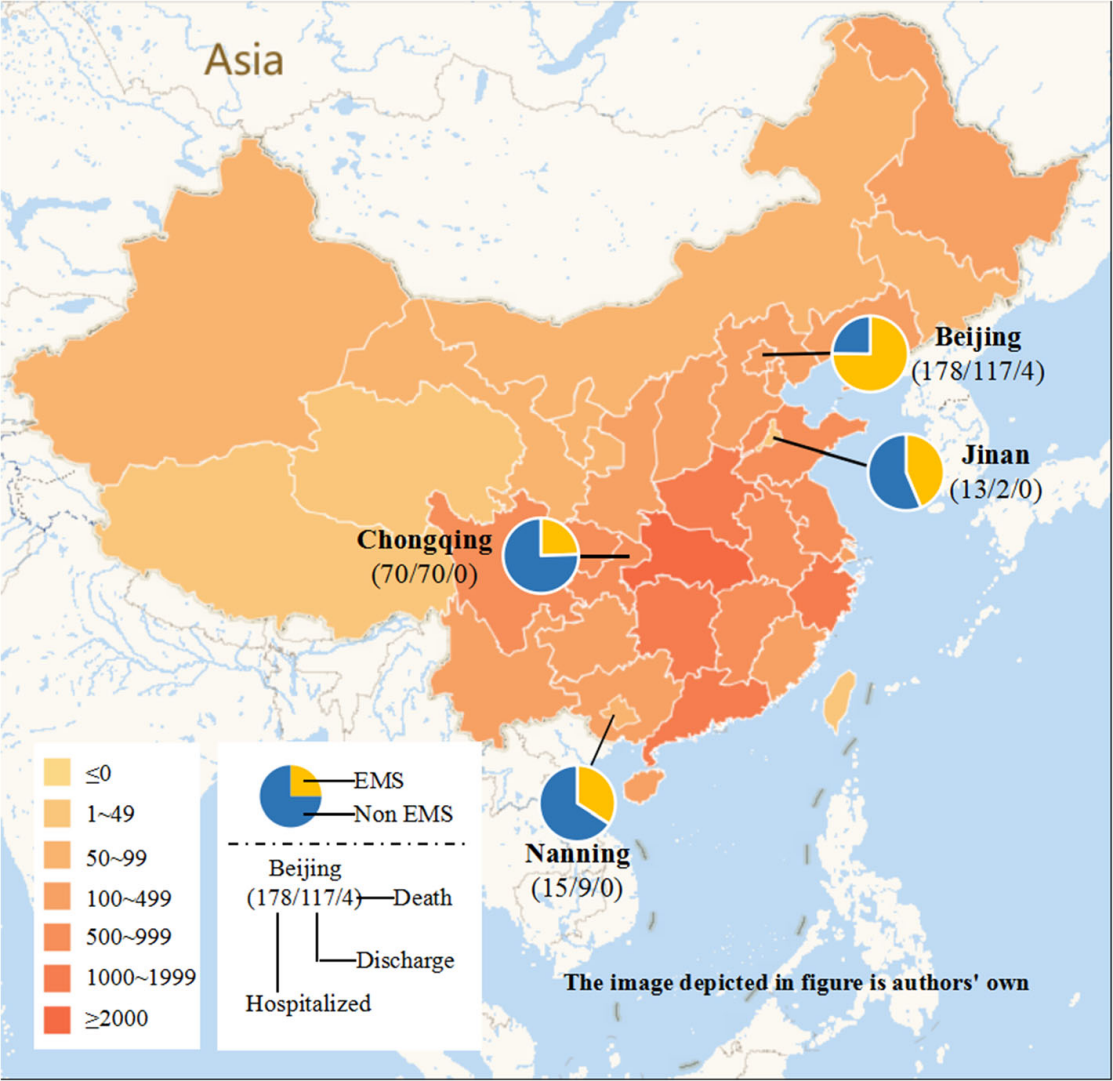
Geographic distribution of COVID-19 infection in China, as of February 21, 2020. EMS: Patient who was transferred by EMS from general hospital to the designated hospital. Non EMS: Patient who didn’t transferred by EMS

From 20 January to 20 February 2020, 478 patients with confirmed COVID-19 infection were enrolled in this study. These patients were transferred from 89 hospitals in Beijing, Chongqing, Jinan, and Nanning to designated COVID-19-receiving hospitals by local EMS (Fig. 1). The mean age of patients was 46.9 years, 88 patients (18.4%) were older than 65 years, and 238 patients (49.8%) were men. The most common symptoms at illness onset were fever (69.7%), cough (47.5%), fatigue (24.5%), dyspnea (8.4%), and headache (7.9%). The mean time from contact with a symptomatic case to illness onset was 7.9 days; the mean time from illness onset to hospital presentation was 2.6 days; and the mean time from hospital presentation to laboratory confirmation of COVID-19 was 1.6 days (Table 1).

**Table 1:** Characteristics of the imported and indigenous cases with COVID-19

	All case (***N*** = 478)	Imported (***N*** = 165)	Indigenous (***N*** = 313)	Statistic value	***P***
**Male** (%)	238(49.8)	98(59.4)	140(44.7)	9.295	0.002
**Age group**				2.713	0.258
Median (range),y	46(1–94)	44(1–89)	47(1–94)		
≤ 18	32(6.7)	9(5.5)	23(7.3)		
19–64	358(74.9)	131(79.4)	227(72.5)		
≥ 65	88(18.4)	25(15.2)	63(20.1)		
**City**				18.564	< 0.001
Beijing	299(62.6)	123(74.5)	176(56.2)		
Chongqing	140(29.3)	33(20.0)	107(34.2)		
Jinan	15(3.1)	6(3.6)	9(2.9)		
Nanning	24(5.0)	3(1.8)	21(6.7)		
**Spectrum of Disease**				10.149	0.006
Asymptomatic	48(10)	8(4.8)	40(12.8)		
Mild	347(72.6)	133(80.6)	214(68.4)		
Severe	83(17.4)	24(14.5)	59(18.8)		
**Symptoms**					
Fever	333(69.7)	136(82.4)	197(62.9)	19.411	< 0.001
Highest temperature				23.665	< 0.001
< 37.3	145(30.3)	29(17.6)	116(37.1)		
37.3–38.0	177(37.0)	74(44.8)	103(32.9)		
38.1–39.0	142(29.7)	53(32.19)	89(28.4)		
> 39.0	14(2.9)	9(5.5)	5(1.6)		
Cough	227(47.5)	75(45.5)	152(48.6)	0.418	0.518
Fatigue	117(24.5)	45(27.3)	72(23.0)	1.065	0.302
Dyspnoea	40(8.4)	11(6.7)	29(9.3)	0.951	0.329
Headache	38(7.9)	12(7.3)	26(8.3)	0.158	0.691
Respiratory rate^a^	20.4 ± 7.2	19.6 ± 3.3	20.9 ± 8.6	−2.322	0.021
**Cluster cases**	328(68.6)	83(50.3)	245(78.3)	39.258	< 0.001
Family	254(53.1)	66(40.0)	188(60.1)		
Other	74(15.5)	17(10.3)	57(18.2)		
**History of Contact**	435(91.0)	165(100.0)	270(86.3)	24.908	< 0.001
Have been to Wuhan in 14 days	136(28.5)	136(82.4)	0(0.0)	360.580	< 0.001
Have contacted the confirmed case in 14 days	285(59.6)	30(18.2)	255(81.5)	179.761	< 0.001
**Outcomes**				29.168	< 0.001
Hospitalized	276(58.8)	68(41.2)	208(66.5)		
Discharged	198(41.4)	96(58.2)	102(32.6)		
Death	4(0.8)	1(0.6)	3(1.0)		

^a^ Mann-Whitney U test

### COVID-19 severity and outcome

**Fig. 2:**
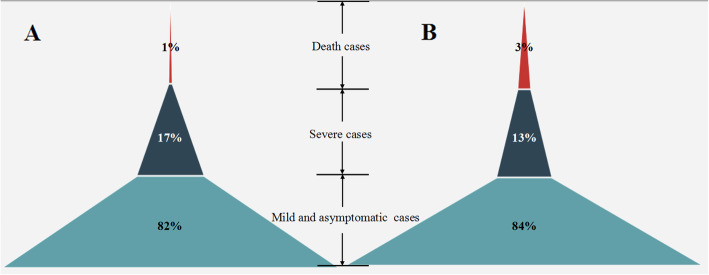
Pyramid of COVID-19 of enrolled in this study (**a**) and nationwide with public data (**b**)

In total, 347 (72.6%) patients had mild disease, 48 (10%) were asymptomatic, and 83 (17.4%) had severe disease. Four (0.8%) patients died. The frequencies of fatal, severe, and mild or asymptomatic COVID-19 per 100 patients were 1, 17 and 82 cases, respectively. Compared with national data (84% mild infection, 13% severe infection, and 3% fatal infection), these results were not significantly different (Z = -1.027, *P* = 0.304). The severity pyramid ratio for all confirmed cases of COVID-19 infection was 1:4:28 (fatal:severe:mild disease) in China (Fig. 2).

Infection of 328 (68.6%) patients was associated with clustering of at least two confirmed cases within a family, hospital, or other location of close contact within 14 days prior to symptom onset. Infection of 254 (53.1%) patients occurred within family clusters.

By the end of 20 February 2020, 198 (41.4%) patients had been discharged, 276 patients (57.7%) remained hospitalized, and the remaining four patients (0.8%) had died.

### Imported versus indigenous cases of COVID-19

**Fig. 3:**
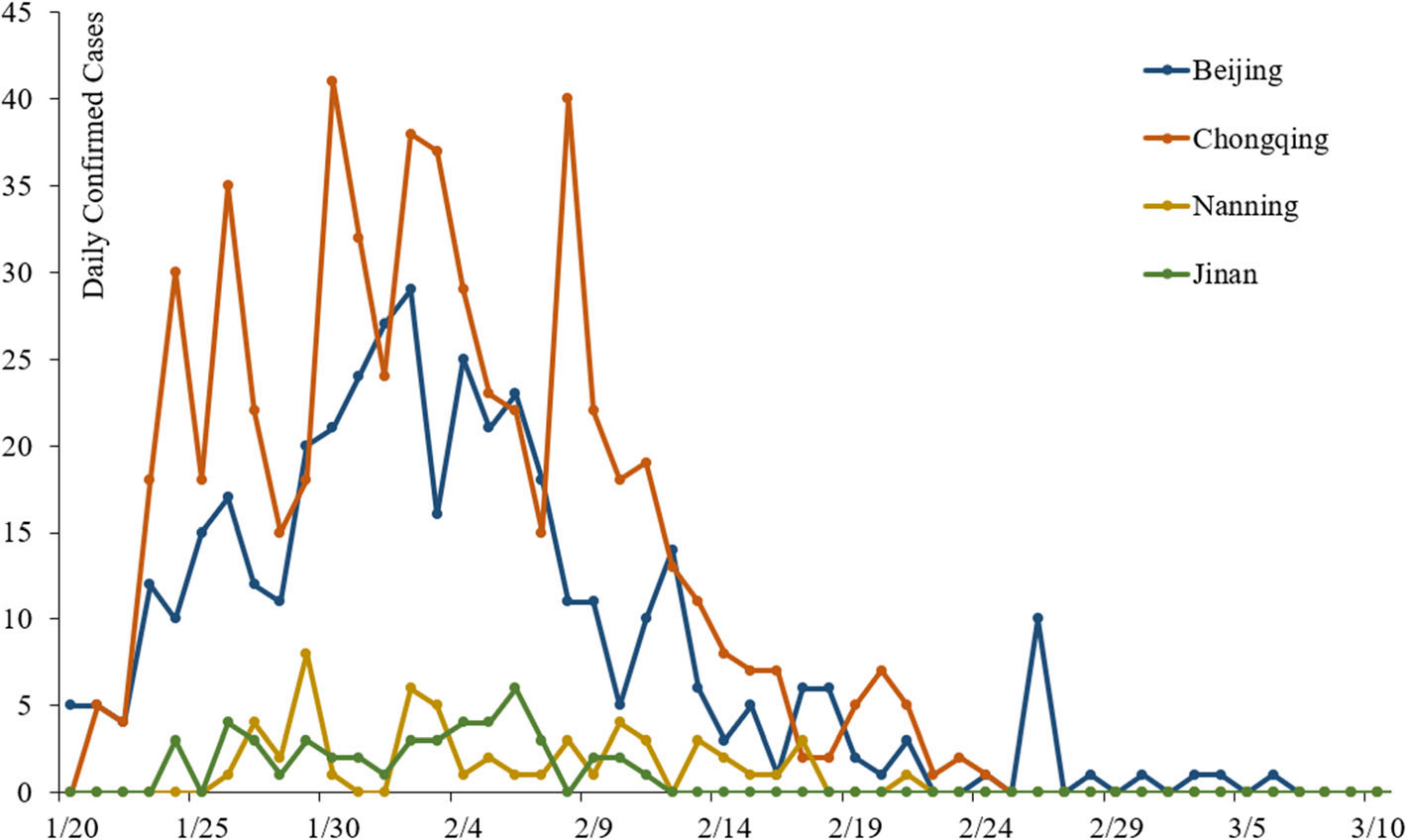
Daily new COVID-19 Cases reported

**Fig. 4:**
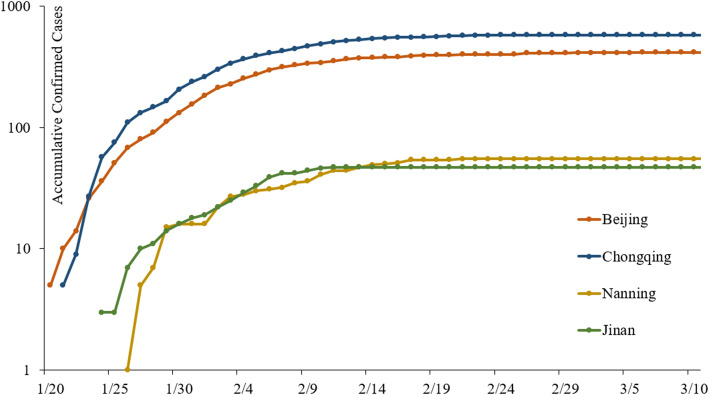
Accumulative COVID-19 Cases reported

**Fig. 5:**
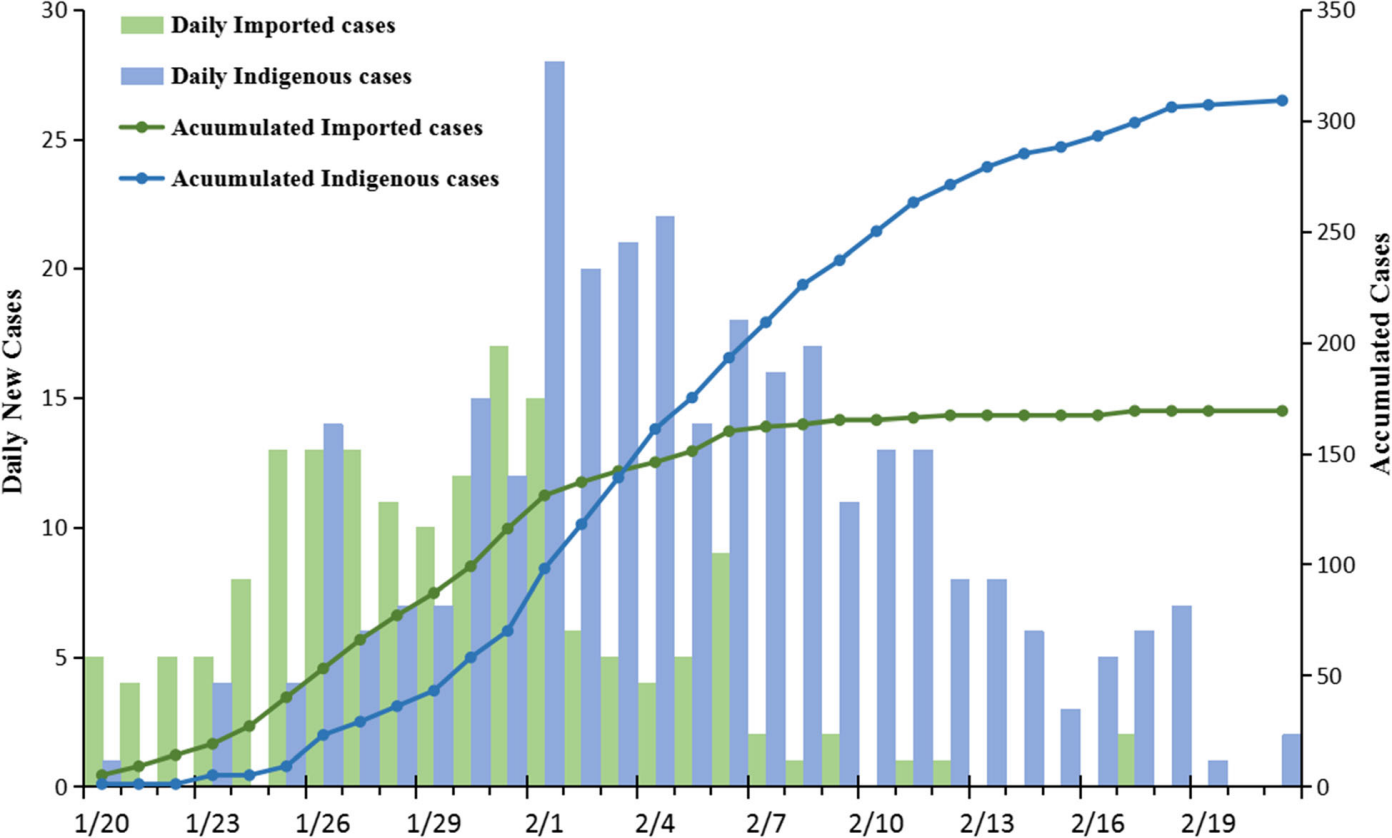
Change of imported cases

The first cases of COVID-19 were diagnosed on 20 January 2020 in Beijing and Chongqing; these patients had traveled to Wuhan before illness onset. Most cases (313, 65.5%) were classified as indigenous cases, while 165 (34.5%) cases were classified as imported cases. The daily number of new imported cases increased during the early stages of the outbreak, exceeding the number of indigenous cases from 20 January to 31 January 2020, then declining after this date (Figs. 3 and 4). The cumulative numbers of both imported and indigenous cases were equivalent on 3 February 2020. The daily numbers of new and cumulative indigenous cases exceeded those of imported cases after 3 February 2020 and declined quickly after 4 February 2020 (Fig. 5).

Compared with indigenous cases, imported cases differed significantly in terms of sex (χ^2^ = 9.295, *P* = 0.002), severity of disease (χ^2^ = 10.149, *P* = 0.006), occurrence of fever (χ^2^ = 19.411, *P* < 0.001), highest body temperature (χ^2^ = 23.665, *P* < 0.001), family clustering (χ^2^ = 39.258, *P* < 0.001), history of contact (χ^2^ = 24.908, *P* < 0.001), and primary outcome (χ^2^ = 29.168, *P* < 0.001).

## Discussion

The goal of this multicenter study was to explore the epidemiological and clinical characteristics of imported COVID-19 cases in four cities in China (Beijing, Chongqing, Jinan, and Nanning) outside of Wuhan. We analyzed data from 478 patients who were transferred by EMS to designated hospitals for treatment of COVID-19. According to predictions by Prof. Wu and colleagues, Chongqing and Beijing were the two cities at highest risk of epidemics caused by imported COVID-19 cases, with Jinan and Nanning being at relatively low risk [16]. Therefore, patients in these four cities were enrolled in this study. As of 20 February 2020, 198 (41.4%) of 478 enrolled patients had been discharged, 276 (57.7%) remained hospitalized, and 4 (0.8%) patients had died. The COVID-19 fatality rate observed in our study was significantly lower than that reported in other studies [7, 9–11]. The ratio of severe to mild and asymptomatic cases was 1:5, and asymptomatic cases were more frequent compared with our previous study [17]. Based on our results, we generated a pyramid of COVID-19 severity. The concept of the COVID-19 pyramid was first proposed by Munster et al. in February 2020 [18] to describe the proportion of mild and asymptomatic cases versus severe cases and fatalities. This pyramid could be used as an intuitive guide to understanding the scope of each type of COVID-19 manifestation [19]. Asymptomatic cases (10%) were mainly observed among close contacts during medical observation in this study. If such individuals are not identified, asymptomatic patients can transmit the virus [20, 21]. Thus, identifying and controlling asymptomatic cases is an important measure to prevent transmission of SARS-CoV-2.

Many of the patients enrolled in this study were young and middle-aged, the mean age was 46.9 years, and only 18.4% of patients were older than 65 years. Roughly half of patients (238, 49.8%) were men. As described in previous studies, the most common symptoms at illness onset were fever, cough, fatigue, and headache, while patients who developed severe disease often presented with dyspnea. Compared with indigenous cases, imported cases differed significantly in terms of sex composition, disease severity, occurrence of fever, peak body temperature, family clustering, history of contact, and primary outcome. More imported patients were men, which suggests that men may travel more frequently than women. While the frequency of asymptomatic cases was higher among indigenous cases compared with imported cases, asymptomatic cases were mainly found among close contacts of symptomatic patients during medical observation. This finding highlights the fact that close contacts should be quarantined and observed for at least 14 days. Fever was significantly more frequent among imported cases compared with indigenous cases, with 37.1% of indigenous cases being afebrile. Most indigenous cases were the result of clustering (78.3%), mainly in family clusters, which indicates that infected family members should be quarantined as early as possible. With globalization and increased international movement made possible by convenient transportation, transmission of SARS-CoV-2 has become an important global public health issue. Thus, strict prevention and control strategies for imported cases from epidemic areas and for exported cases to other regions are vital. In this study, we found that 34.5% cases were imported from Wuhan or other cities in Hubei Province. The case load transitioned over time to local indigenous infections. The daily number of new imported cases increased during the early stages of the epidemic, exceeding the number of indigenous cases for about 10 days before declining. Thereafter, numbers of daily new and cumulative indigenous cases exceeded those of imported cases until both declined quickly after 4 February 2020. Therefore, all four cities were highly successful in preventing and controlling the COVID-19 outbreak. These cities have greatly benefited from strong leadership and from previous experience with the SARS-CoV outbreak in 2003.

This study had several limitations. First, only COVID-19 confirmed cases transferred by EMS were included; patients directly admitted to the designated COVID-19 hospitals were not enrolled. The study would have benefited from covering as wide a population as possible. Second, the observation period of this study was relatively short at 30 days, and many COVID-19 patients require prolonged observation times. However, we successfully captured the characteristics of the middle stages of the COVID-19 epidemic in four cities in China, during the transition from imported to indigenous cases. These data may be of value for future outbreak control.

## Conclusions

In this study, we analyzed the characteristics of imported cases of COVID-19 in four cities in China outside of Wuhan. Within the population studied, imported cases had distinct characteristics from those of indigenous cases, with lower fatality rates and higher discharge rates. New infections shifted from imported cases to local infection gradually, and overall infections have declined to a low level. We suggest that preventing import of cases and controlling spread within local areas can help prevent SARS-CoV-2 infection spread.

## Data Availability

The datasets used and/or analysed during the current study are available from the corresponding author on reasonable request.

## Abbreviations

CDC: Center for Disease Control
COVID-19: Corona Virus Disease 2019
EMS: Emergency Medical Service
WHO: World Health Organization
